# Development of a Novel Prognostic Model for Predicting Lymph Node Metastasis in Early Colorectal Cancer: Analysis Based on the Surveillance, Epidemiology, and End Results Database

**DOI:** 10.3389/fonc.2021.614398

**Published:** 2021-03-25

**Authors:** Ji Hyun Ahn, Min Seob Kwak, Hun Hee Lee, Jae Myung Cha, Hyun Phil Shin, Jung Won Jeon, Jin Young Yoon

**Affiliations:** Department of Internal Medicine, Kyung Hee University Hospital at Gangdong, Kyung Hee University College of Medicine, Seoul, South Korea

**Keywords:** machine learning, colorectal cancer, prediction, metastasis, model

## Abstract

**Background:**

Identification of a simplified prediction model for lymph node metastasis (LNM) for patients with early colorectal cancer (CRC) is urgently needed to determine treatment and follow-up strategies. Therefore, in this study, we aimed to develop an accurate predictive model for LNM in early CRC.

**Methods:**

We analyzed data from the 2004-2016 Surveillance Epidemiology and End Results database to develop and validate prediction models for LNM. Seven models, namely, logistic regression, XGBoost, k-nearest neighbors, classification and regression trees model, support vector machines, neural network, and random forest (RF) models, were used.

**Results:**

A total of 26,733 patients with a diagnosis of early CRC (T1) were analyzed. The models included 8 independent prognostic variables; age at diagnosis, sex, race, primary site, histologic type, tumor grade, and, tumor size. LNM was significantly more frequent in patients with larger tumors, women, younger patients, and patients with more poorly differentiated tumor. The RF model showed the best predictive performance in comparison to the other method, achieving an accuracy of 96.0%, a sensitivity of 99.7%, a specificity of 92.9%, and an area under the curve of 0.991. Tumor size is the most important features in predicting LNM in early CRC.

**Conclusion:**

We established a simplified reproducible predictive model for LNM in early CRC that could be used to guide treatment decisions. These findings warrant further confirmation in large prospective clinical trials.

## Introduction

Colorectal cancer (CRC) is a major cause of morbidity and mortality worldwide, its importance is expected to continue increasing over time (1, 2). In recent years, increased awareness and the introduction of population-based surveillance and screening programs have led to achieving higher rates of precancerous dysplastic lesions or early CRC detection (3, 4).

Early CRC is a tumor that is confined to the mucosa and/or submucosa regardless of the presence of regional lymph node metastasis (LNM). In certain cases of early CRC, endoscopic resection is a less invasive and cost-effective treatment compared to surgery (5–7). However, the CRC patients with LNM or distant metastasis cannot be adequately cured by local endoscopic treatment alone, and therefore subsequently require surgical resection for achieving a curative treatment.

LNM is found in approximately 6–16% of the patients with submucosal invasive CRC (8–10), however, the number might be underestimated, as clinicians make important treatment decisions based on limited examinations, such as computed tomography (CT) and ultrasonography.

Thus, an accurate and fast assessment of locoregional and/or distant metastases in patients with early CRC is essential to determine whether these patients should undergo additional surgical resections or be needed surveillance regularly. Currently, no universally accepted indications and criteria exist for additional surgical resection after endoscopic resection, even though a fast and accurate assessment of the risk of locoregional LNM after local endoscopic treatment of patients with early CRC is necessary.

Therefore, the aim of present study was to develop a novel prediction model for LNM by using simple histopathological and clinical parameters with high reliability, that can be used to improve patient risk stratification in early CRC.

## Materials and Methods

### Data Source

This study used the Surveillance, Epidemiology, and End Results (SEER) Program database from the National Cancer Institute, which is publicly available U.S. cancer registries. The registry collects and publishes cancer incidence, mortality, and survival data from 17 population-based cancer registries, covering approximately 34.6% of the U.S. population (Iowa, Los Angeles, Connecticut, Utah, Greater California, Idaho, Georgia Center for Cancer Statistics, San Francisco-Oakland, San Jose-Monterey, Louisiana, Hawaii, Massachusetts, Alaska Native tumor registry, Kentucky, New Mexico, New York, Seattle-Puget Sound) (11). The database is roughly represent the U.S. population and includes information on over 9 million cancer cases with over 550,000 new cases added to the database annually. It offers a powerful resource for researchers focused on understanding the natural history of CRC and improving quality healthcare for the patients (11, 12). This retrospective cohort study was evaluated and approved by the Institutional Review Board of the Kyung Hee University Hospital at Gangdong (KHNMC IRB 2020-01-015).

### Study Population

**Figure 1:**
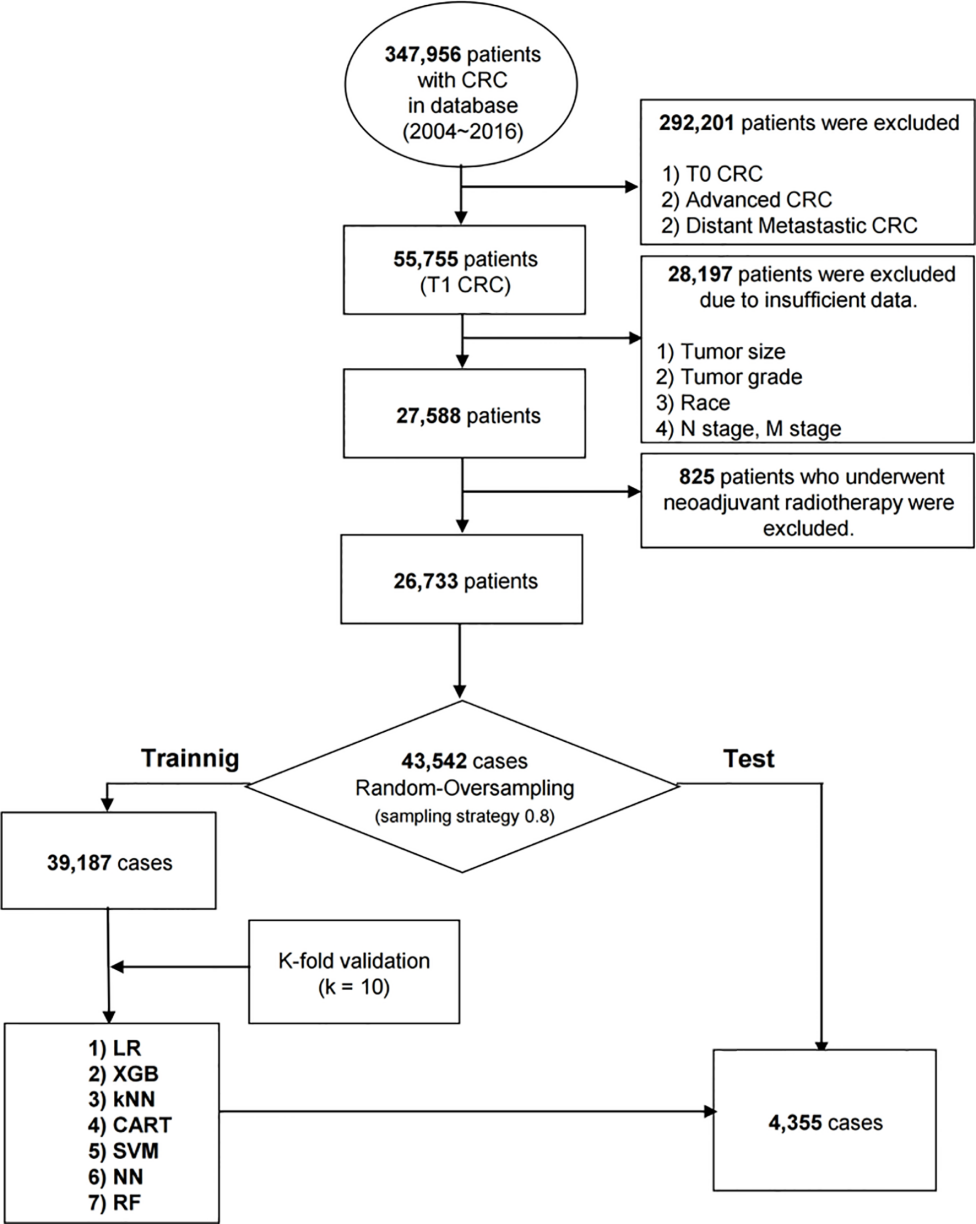
The Workflow of the development process.

The SEER registry collects data including age at diagnosis, sex, race, primary site, histologic type, tumor grade, tumor size, and tumor depth. Using the SEER 1975–2016 database (released 4/15/2019), we analyzed data from all patients diagnosed with T1 CRC for the years 2004-2016. T1 CRC was defined as infiltration of the tumor into the submucosa. We extracted clinical demographic data, including age at diagnosis, sex, race and tumor information including location, size, grade, histologic type, and American Joint Committee on Cancer 7th TNM stages by using SEER disease codes. Tumor location was determined by using the following codes: C18.0 (cecum); C18.1 (appendix); C18.2 (ascending colon); C18.3 (hepatic flexure); C18.4 (transverse colon); C18.5 (splenic flexure); C18.6 (descending colon); C18.7 (sigmoid colon); C18.8 (overlapping lesion of colon); C18.9 (colon); rectosigmoid (C19.9); and rectum (C20.9). The morphology of cancer was categorized according to the ICD-0-3 histology and behavior codes: 8010/3, (carcinoma, NOS); 8020/3, (carcinoma, undifferentiated, NOS); 8140/3, (adenocarcinoma, NOS); 8144/3, (adenocarcinoma, intestinal type); 8210/3, (adenocarcinoma in adenomatous polyp); 8211/3, (tubular adenocarcinoma); 8255/3, (adenocarcinoma with mixed subtypes); 8261/3, (adenocarcinoma in villous adenoma); 8262/3, (villous adenocarcinoma); 8263/3, (adenocarcinoma in tubulovillous adenoma); 8440/3, (cystadenocarcinoma, NOS); 8470/3, (mucinous cystadenocarcinoma, NOS); 8480/3, (mucinous adenocarcinoma); 8481/3, (mucin-producing adenocarcinoma); 8490/3, (signet ring cell carcinoma); and8221/3, (adenocarcinoma in multiple adenomatous polyps). For tumor differentiation grading, we used a four tier classification including well differentiated, moderately differentiated, poorly differentiated, undifferentiated, which is proposed by WHO grading system (13). In order to exclude potentially confounding factor, the patients who received preoperative radiation treatment were excluded. The overall scheme of the workflow is illustrated in **Figure 1**.

### Establishment of the Predictive Model

In this study, we used seven machine-learning (ML) models that are commonly used to predict LNM in patients with early CRC. For the linear model, the logistic regression model (LR) was selected (14). The neural network model (NN), which is one of the important classes of nonlinear prediction models and has been reported in a previous study was used (15). For the kernel-based model, we applied the support vector machine (SVM), which is adopted in many clinical applications (16). For the decision tree approach, the classification and regression trees model (CART), XGBoost (XGB) model and random forest (RF) model, which have also been used in clinical research were included (17–19). Finally, for the basic prediction technique, k-nearest neighbor algorithm (kNN) was selected (20).

We used random oversampling method to improve the classifier performance for the minority classes in our imbalanced classes (21). First, the patients were randomly assigned to a training set (90%) and a test set (10%), where the two class (LNM group vs. non-LNM group) proportions in each set were the same. In the training set, we performed k-fold cross-validation (k = 10), and grid search was used to find the best parameter combinations. For each set of parameters, we fitted the model in turn with 9/10 of data and used 1/10 of data for validation.

### Assessment of Prediction Models

To ensure a fair comparison of the models, we used the confusion matrix, area under the curve (AUC), sensitivity (recall), specificity, accuracy, average precision (AP), false positive rate, and precision as performance indicators. We used the AU-ROC as the performance index and the AP value as the criterion for the precision-recall (PR) curve (22). The average value of the parameter was finally executed on the test set.

### Statistical Analysis

All data were obtained using the SEER*Stat software (8.3.6 version; Surveillance Research Program, National Cancer Institute). All analyses were performed with Python (version 3.6.9) and R statistical software (version 3.6.0). Demographic differences between the two groups were tested using the Student’s t-test and Pearson chi‐square test. To better evaluate the performance of the models, we used a paired t test to compare the AU-ROC further in each resampling calculation. A two‐sided *P ≤ 0.05* was considered statistically significant.

## Results

### Baseline Characteristics

A total of 347,956 patients with CRC between 2004 and 2016 were collected, of which about 292,201 patients were excluded from the study because they were diagnosed with T0 or advanced CRC with or without distant metastasis. After excluding 28,197 patients with insufficient data and 825 patients treated with preoperative radiation therapy, 26,733 patients with a diagnosis of early CRC (T1) were analyzed. The model included eight independent prognostic variables, including age at diagnosis, sex, race, primary site, histologic type, tumor grade, and, tumor size. The analyzed patients were divided into the LNM (2,543 patients, 9.5%) and non-LNM groups (24,190 patients, 90.5%). The younger people (< 60 years) tended to have more LNM at diagnosis compared with the older group (*P* < 0.001). Significantly higher LNM in women compared with men was observed in the patients with early CRC (*P* < 0.001). The proportion of LNM in the distal colon included the descending colon, sigmoid colon, and the rectosigmoid junction, was significantly higher than that in the colon proximal to the splenic flexure (*P* < 0.001). The overall racial and/or ethnic distribution was 69.7% non-Hispanic whites, 11.9% non-Hispanic blacks, 9.0% Hispanics, 8.9% non-Hispanic Asians or Pacific Islander, and 0.5% others (non-Hispanic American, Indian, Alaska natives). Among all patients evaluated, 20.8% (n=5,572) had well differentiated tumor; 71.2% (n=19,026), moderately differentiated; 7.1% (n=1,902), poorly differentiated; 0.9% (n=233), undifferentiated cancer. The mean tumor size was significantly larger in the early CRC patients with LNM than in those of without LNM (22.8mm vs. 20.6 mm) (*P* < 0.001). **Table 1** shows the overall distribution of baseline characteristics of the study population.

**Table 1:** Baseline characteristics.

Variables	LNM (-)	LNM (+)	
	N = 24190	N = 2543	*P*-value
Age at diagnosis, n (%)			<0.001
0-9	0 (0.0)	1 (0.0)	
10-19	5 (0.0)	1 (0.0)	
20-29	73 (0.3)	10 (0.4)	
30-39	339 (1.4)	61 (2.4)	
40-49	1511 (6.3)	241 (9.5)	
50-59	5684 (23.5)	730 (28.7)	
60-69	6775 (28.0)	683 (26.9)	
70-79	5952 (24.6)	544 (21.4)	
80-89	3410 (14.1)	245 (9.6)	
90-99	441 (1.8)	27 (1.1)	
Sex, n (%)			<0.001
M	12864 (53.2)	1254 (49.3)	
F	11326 (46.8)	1289 (50.7)	
Primary site, n (%)			<0.001
Cecum	3355 (13.9)	381 (15.0)	
Appendix	119 (0.5)	4 (0.2)	
Ascending colon	3493 (14.4)	300 (11.8)	
Hepatic flexure of colon	665 (2.7)	61 (2.4)	
Transverse colon	1545 (6.4)	119 (4.7)	
Splenic flexure of colon	381 (1.6)	39 (1.5)	
Descending colon	1009 (4.2)	97 (3.8)	
Sigmoid colon	6193 (25.6)	773 (30.4)	
Overlapping lesion of colon	78 (0.3)	5 (0.2)	
Colon, NOS	111 (0.5)	7 (0.3)	
Rectosigmoid junction	1737 (7.2)	268 (10.5)	
Rectum, NOS	5504 (22.7)	489 (19.2)	
Tumor grade, n (%)			<0.001
Well differentiated	5284 (21.8)	288 (11.3)	
Moderately differentiated	17173 (71.0)	1853 (72.9)	
Poorly differentiated	1538 (6.4)	364 (14.3)	
Undifferentiated	195 (0.8)	38 (1.5)	
Race, n (%)			<0.001
Hispanic	2186 (9.1)	228 (9.0)	
Non-Hispanic American Indian/Alaska Native	129 (0.5)	10 (0.4)	
Non-Hispanic Asian or Pacific Islander	2099 (8.7)	270 (10.6)	
Non-Hispanic Black	2837 (11.7)	354 (13.9)	
Non-Hispanic White	16939 (70.0)	1681 (66.1)	
Tumor type, n (%)			<0.001
Carcinoma, NOS	40 (0.2)	6 (0.2)	
Carcinoma, undifferentiated, NOS	1 (0.0)	1 (0.0)	
Adenocarcinoma, NOS	9657 (39.9)	1148 (45.1)	
Adenocarcinoma, intestinal type	2 (0.0)	2 (0.1)	
Adenocarcinoma in adenomatous polyp	5943 (24.6)	513 (20.2)	
Tubular adenocarcinoma	47 (0.2)	2 (0.1)	
Adenocarcinoma with mixed subtypes	20 (0.1)	4 (0.2)	
Adenocarcinoma in villous adenoma	1378 (5.7)	130 (5.1)	
Villous adenocarcinoma	27 (0.1)	1 (0.0)	
Adenocarcinoma in tubulovillous adenoma	6420 (26.5)	614 (24.2)	
Cystadenocarcinoma, NOS	1 (0.0)	0 (0.0)	
Mucinous cystadenocarcinoma, NOS	11 (0.1)	0 (0.0)	
Mucinous adenocarcinoma	487 (2.0)	82 (3.2)	
Mucin-producing adenocarcinoma	107 (0.4)	20 (0.8)	
Signet ring cell carcinoma	49 (0.2)	20 (0.8)	
Tumor size, mm, mean (SD)	20.6 (25.4)	22.8 (20.9)	<0.001

SD, standard deviation.

### Tuning of Parameters

We trained the SVM a combination of a C value of 1.0 and a kernel smoothing parameter σ of 0.001. For kNN, a relatively large number of k = 14 was optimal. XGB was performed using the parameters with a maximum depth of 6 and a minimum child weight of 1. For NN, the hyper-parameters were changed during training to obtain the optimal model based on the validation set. The final selected hyper-parameters were a learning rate of 0.001, epoch of 300, hidden layer of 3, dropout rate of 0.3, and batch size of 128. For RF, a relatively large number of randomly selected 61 subtrees provided the best performance.

### Performance of Developed Models

**Figure 2:**
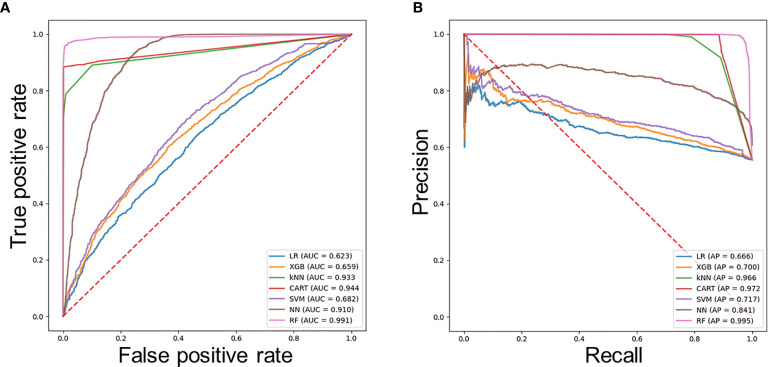
Evaluation of the predictive models. **(A)** Average ROC curves of seven models. **(B)** Average PR curves, indicating the tradeoff between precision and recall.

The average ROC curves and PR curves during the training are shown in **Figure 2**. Most models had AUC values above 0.81, but the values of LR, XGB, and SVM were lower. The confusion matrix was also calculated for the seven models (**Table 2**). As shown in **Table 2**, LR, XGB, and SVM generated a large number of FNs, and kNN and CART models had a large number of FPs during the prediction process. The RF model produced the minimum number of FN (= 5) and FP (= 171). **Table 3** shows the AUC, sensitivity, specificity, precision, negative predictive value (NPV), false discovery rate (FDR), accuracy, AP, F1, and Matthews correlation coefficient of each model. The linear model LR showed the worst performance; its accuracy rate was up to 0.60, whereas the accuracy of RF was up to 0.96.

**Table 2:** Confusion matrices of developed models.

Confusion matrix			
	Actual	Prediction	
		LNM (-)	LNM (+)
LR	LNM (-)	1903	516
	LNM (+)	1240	696
XGB	LNM (-)	2163	256
	LNM (+)	1468	468
kNN	LNM (-)	1907	512
	LNM (+)	18	1918
CART	LNM (-)	1907	512
	LNM (+)	18	1918
SVM	LNM (-)	1898	521
	LNM (+)	1053	883
NN	LNM (-)	1995	424
	LNM (+)	304	1632
RF	LNM (-)	2248	171
	LNM (+)	5	1931

LR, logistic regression; XGB, XGBoost, kNN, k-nearest neighbor; CART, classification and regression trees model; SVM, support vector machine; NN, neural network; RF, random forest.

**Table 3:** Performance of developed models.

	AUC	Sensitivity	Specificity	Precision	NPV	FDR	Accuracy	AP	F1 Score	Matthews correlation coefficient
Models										
LR	0.623	0.360	0.787	0.574	0.606	0.426	0.597	0.666	0.442	0.162
XGB	0.659	0.242	0.894	0.646	0.596	0.354	0.604	0.700	0.352	0.181
kNN	0.933	0.991	0.788	0.789	0.991	0.211	0.878	0.966	0.879	0.780
CART	0.944	0.991	0.788	0.789	0.991	0.211	0.878	0.972	0.879	0.780
SVM	0.682	0.456	0.785	0.629	0.643	0.371	0.639	0.717	0.529	0.256
NN	0.910	0.843	0.825	0.794	0.868	0.206	0.833	0.841	0.818	0.665
RF	0.991	0.997	0.929	0.919	0.998	0.081	0.960	0.995	0.956	0.922

AUC, area under curve; NPV, negative predictive value; FDR, false discovery rate; AP, average precision; LR, logistic regression; XGB, XGBoost, kNN, k-nearest neighbor; CART, classification and regression trees model; SVM, support vector machine; NN, neural network; RF, random forest.

The accuracy of the other models was less than 0.90. RF achieved the highest AUC value of 0.991, and CART had an AU-ROC value of 0.944. LR had the lowest AUC value of 0.623. The RF model showed the best sensitivity and specificity, as well as the best precision, NPV, FDR, accuracy, AP score, F1 score and Matthews correlation coefficient value.

### Feature Importance Comparisons between Algorithms

**Figure 3:**
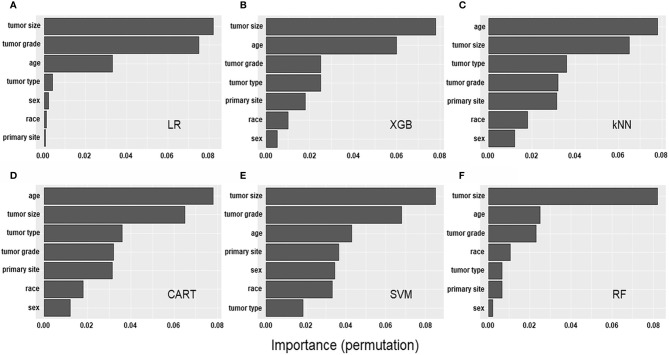
Factor importance of the developed models. The **(A–F)** Bar graphs describe the proportion of importance of the different predictors in the model.

We quantified the variable importance using the coefficients of permutation importance for LNM in each model (**Figure 3**). For most of the models, the variables including tumor grade, depth of tumor, and age had important influences on the predictability for LNM in early CRC. Based on our quantification, tumor size showed the highest frequency for the top predictors in four of the six models.

## Discussion

In this study, we established a novel predictive model by combining eight clinicopathologic parameters to predict LNM in early CRC using seven ML models. To the best of our knowledge, this is the first large-scale study to develop a predictive model for LNM by combining easily available simple clinical and pathological data in patients with early CRC. Clinicians are often confronted with the difficulty of selecting candidates who will benefit from surgery after local endoscopic resection.

Currently, in clinical practice, risk stratification in these patients is usually performed by histopathologists carefully analyzing the specimen to determine the risk of LNM, caused by the limited capacity of CT to accurately identify LNM (23).

In previous studies, the pathological factors that showed the strongest independent predictive value for LNM in early CRC are tumor type, poor histological differentiation, and the depth of submucosal invasion (24–27). However, the high interobserver variability in the pathological assessment limits their clinical usefulness and should therefore be interpreted with caution as a univariate marker when deciding whether to proceed with surgery (28, 29). Therefore, the multivariable risk model combining the histopathological data with clinical data can reduce the inaccuracies associated with relying on individual subjective markers and to better define the optimal treatment strategy for early CRC.

With the recent rapid development of computer-aided technology, the application of ML model in cancer diagnosis has an important role; it is being widely used in the medical field with growing trend toward predictive medicine (30–32). We hereby developed an ML model by using the simple clinicopathological parameters in large data, which provided high predictive ability of LNM for patients with early CRC.

To date, a few ML models for prediction of metastasis in patients with early CRC have been developed and evaluated for prognosis and prediction in patients with early CRC (33–36). Ichimasa et al. developed the SVM model with 45 clinicopathologic factors for prediction of LNM in patients with early CRC. They reported that artificial intelligence significantly reduces unnecessary extra surgery after endoscopic resection of T1 CRC without LNM positive in comparison to the current guidelines (33). Another Japanese study showed a deep learning model for predicting LNM from pathology images with cytokeratin immunohistochemistry in early CRC (34). However, these studies were retrospective in nature with single center or small numbers of patients. Due to the low rate of metastasis in early CRC, only a limited number of events exist, leading to limited data. Furthermore, inadequate data could not provide sufficient satisfactory performance under ML algorithms and may have led to lower predictive performance ranging from 0.821 to 0.913, which is less than the result from our RF model. A recent Chinese study also presented a predicting model for LNM that incorporates both the radiomics signature, which combine multiple individual CT imaging features, and several clinical factors using the multivariable logistic regression analysis (35). Although this might be an interesting attempt, the model validity is not guaranteed considering the heterogeneity in the quality of CT image between facilities and its accuracy of approximately 78%, which is lower than the performance of the predictive model we constructed. Lastly, Kudo et al. also employed deep-learning-based modeling to predict LNM in T1 CRC (36). However, they only used NN model for nonlinear dynamic system with smaller sample size than our study and assessed LNM using only CT imaging in the cases treated by endoscopic resection, because pathologic confirmation was not available.

Meanwhile, the reason why the RF model outperforms the other ML algorithms is not easily explained. It might be attributed to that the RF models generally demonstrate the most substantial improvement over linear methods and, might be outperform kernel-based model and neural network model in many categorical variables and some outliers from the nature of large retrospective cohort data. However, to build robust prognostic models for LNM in early CRC, other variables, such as gene expression and histologic image data beyond clinical-pathological variables, should be needed.

In our study, we investigated the variable importance of the predictive models developed, as it could be useful for decision-making by clinicians. Our findings indicated that tumor size was the most important factor for predicting the presence of LNM in early CRC. The prognostic value of tumor size in CRC has long been studied, but no consensus has been reached. Zhang et al. and Kornprat et al. demonstrated a significant association between tumor size and metastasis in CRC (37, 38), whereas Miller et al. indicated no prognostic significance of tumor size in CRC (39). Furthermore, its potential prognostic role in patients with early CRC has not been well investigated. This is the first largest study to identify the prognostic value of tumor size for early CRC and provide statistical evidence for further prospective study. Despite the aforementioned, the current study has several limitations. First, since the SEER database is a nationwide program, several diagnostic criteria, such as histological grades and verification of tumor locations might be subjective, which could cause potential systematic bias. Second, detailed histopathological data, such as lymphovascular invasion, tumor budding, and precise depth of tumor invasion that have been associated with metastasis are insufficient. These data require further assessment to improve the performance of our ML algorithms. Third, our study comprised predominately of white patients; thus, the findings may not be generalized to other racial populations. Finally, the data have a class imbalance problem between the patients with and those without LNM, which means that the rate of LNM is low in early CRC. Therefore, during the tuning process, the parameters had to be further optimizing to avoid overfitting. To further improve the accuracy of the established model, it is necessary to collect more clinical data and further optimizing the parameters are necessary in subsequent studies.

In conclusion, we established and compared seven models to predict metastasis in early CRC by using easily available clinical and histopathological features in real practice. The RF model, a simplified reproducible predictive model, showed the highest predictive power compared with the other models. Tumor size the most important predictor of LNM in early CRC. Therefore, the patients with tumor larger than 3 cm, who were identified as high-risk through the model, may requires careful attention to selection and additional surgical treatment in early CRC. However, because of the limitations inherent in studies based on observational data, these findings should be confirmed in prospective clinical trials.

## Data Availability Statement

Publicly available datasets were analyzed in this study. This data can be found here: https://seer.cancer.gov.

## Author Contributions

MK designed the study. JA, JC and HS analyzed and interpreted the data and wrote the manuscript. JJ and JY supervised the project and revised the paper. All authors contributed to the article and approved the submitted version.

## Funding

This research was supported by the Basic Science Research Program of the National Research Foundation of Korea (NRF), which is funded by the Korean Ministry of Science, ICT and Future Planning [grant number NRF- 2019R1C1C1003524].

## Conflict of Interest

The authors declare that the research was conducted in the absence of any commercial or financial relationships that could be construed as a potential conflict of interest.
